# Remote cerebellar hemorrhage following resection of a supratentorial tumor: a case report

**DOI:** 10.4076/1757-1626-2-7299

**Published:** 2009-06-12

**Authors:** Mehdi Sasani, Ali Fahir Ozer, Tunc Oktenoglu, Ercan Karaarslan, Hadi Sasani, Tuncay Kaner

**Affiliations:** 1 Neurosurgery Department, American HospitalGuzelbahce Sk. No: 20, 34365 Nisantasi - IstanbulTurkey; 2 Radiology DepartmentAmerican HospitalGuzelbahce Sk. No: 20, 34365 Nisantasi - IstanbulTurkey; 3 Istanbul University, Medicine Faculty Radiology DepartmentIstanbulTurkey; 4 Neurosurgery Department, Pendik State HospitalIstanbulTurkey

## Abstract

Remote cerebellar hemorrhage after supratentorial surgery is rare, ranging between 0.08% and 0.29% in adults and children. However, it is extremely rare in children. This phenomenon underlying mechanisms remain obscure. A 14-year-old male child patient had a history of right focal seizures and underwent craniotomy for a left frontal mass (Dysembryoplastic Neuroepithelial Tumor). First hours post recovery period, the patient was somnolent and had right hemiparesis. Postoperative Computer Tomography and magnetic resonance imaging findings revealed that the patient had developed remote cerebellar hemorrhage. He was treated conservatively, and was free of neurological deficits.

Although dehydration and the displacement of the cerebellum are associated with this phenomenon after supratentorial surgery, the identification of the exact etiological factors remains elusive. It is advisable for case givers to be aware of the high potential risk of morbidity and mortality of this entity. Preoperative attention to prevent cerebrospinal fluid overflow leakage and exaggerated dehydration of the patient may prevent remote cerebellar hemorrhages.

## Introduction

Hemorrhage in the area of the operative site is a well known complication of cranial surgery. However, remote cerebellar hemorrhage following supratentorial craniotomy [1], even after spinal surgery [2], is a very infrequent complication. Remote cerebellar hemorrhage is most common between the ages of 30 and 60 years [3]. The underlying pathophysiology of this subset of cerebellar hemorrhage is not clear yet, reviews and reports speculate around a venous origin, but perioperative or, even more likely, postoperative loss of large volumes of cerebrospinal fluid (CSF) seems to be related to remote cerebellar hemorrhage [4].

The case presented here, in which remote cerebellar hemorrhage and massive cerebral and cerebellar edema occurred following resection of Dysembryoplastic neuroepithelial tumor on the left frontal site, is extremely rare and an unusual complication in children.

## Case presentation

**Figure 1.**
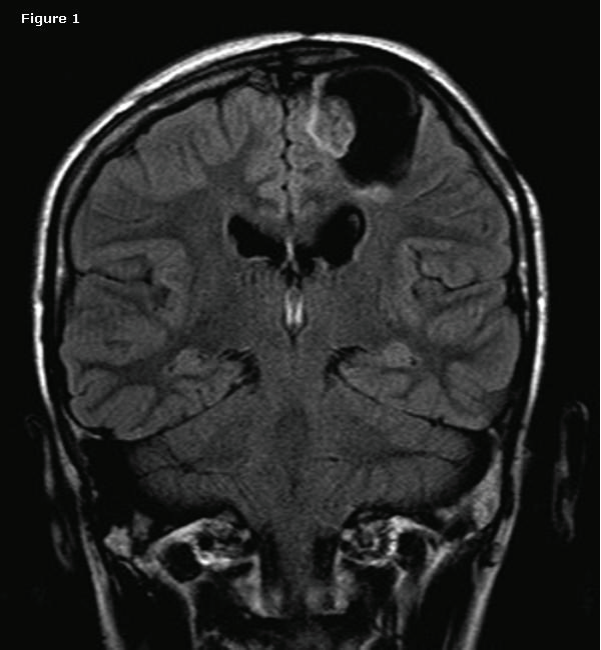
Coronal FLAIR image shows approximately 3 × 4 cm in diameters cystic mass in the left frontal lobe. Cystic mass has a nodulary portion with different signal at the medial wall.

This 14-year-old Turkish male patient from Azerbaijan had a history of right focal seizures that began when he was 3 years of age. His previous examination and therapy was significant in that he experienced episodes of focal seizure until 6 years of age. He was treated with antiepileptic medicine (Phenobarbital), and a cranial MR image showed a left frontal mass. He underwent a craniotomy, and a biopsy was done during the first operation. Histopathological examination revealed a Dysembryoplastic Neuroepithelial Tumor (DNET). His seizures decreased over one year (Phenobarbital was continued for one year), then were lost without treatment of antiepileptic medicine until 12 years of age. He had experienced medically refractory complex partial seizures to sodium valproate with loss of consciousness. His neurological examination found no significant deficits. Magnetic resonance (MR) imaging of the brain showed a cystic mass in the left frontal lobe, directly adjacent to the pre motor area gyrus without gadolinium enhancement (Figure 1). An electroencephalography (EEG) showed an epileptic wave focus on the left frontal lobe. Meanwhile, the patient's coagulation parameters, such as prothrombin time, activated partial thromboplastin time, international normalized ratio and thrombosit count, were within normal limits. The patient underwent a craniotomy for a left frontal tumor resection. Perioperative dehydration was achieved by a diuretic agent (Furosemide, 20 mg/2ml) and the infusion of 20% mannitol (250 ml). The brain was shrunken by the administration of the above agent. The aggressive anti-edema procedure caused the brain to become very slack.

Surgery was completed without complications. An epidural or a subgaleal drainage tube was not used. During surgery, the patient's mean arterial pressure ranged between 60 and 105 mm Hg. Histopathological examination of the surgical specimen yielded findings consistent with Dysembryoplastic neuroepithelial tumor.

Postoperative Course: The patient was transferred directly to ICU after surgery. He was recovered in ICU for six hours postoperatively. First hours post recovery period (7 hours postoperatively), the patient was somnolent and on neurologic examination, had right hemiparesis. The mean arterial pressure was between 55 and 100 mmHg.

**Figure 2.**
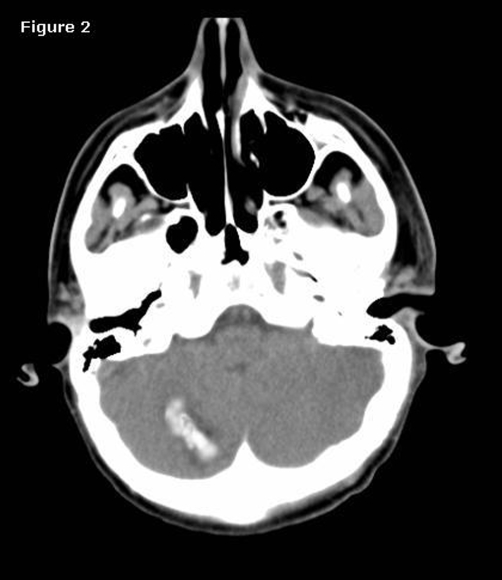
Immediate postoperative CT scans show acute hemorrhage in the right cerebellum fulia.

Immediate postoperative brain computed tomography showed massive edema that caused a disappearance of the resected mass cavity and a suspected linear hemorrhage in the right cerebellar hemisphere in the maximal diameter 15 × 20 mm (Figure 2).

**Figure 3.**
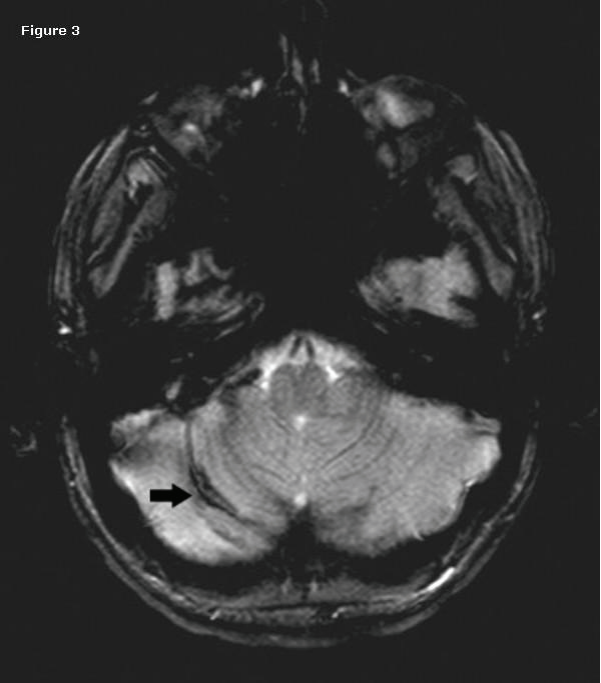
Postoperative first day MRI confirm the hemorrhage. Axial gradient T2 weighted images show a right cerebellar linear hypointensity, compatible with remote cerebellar hemorrhage.

Anti edema treatment was achieved with 20% mannitol (100 ml infusion per six hours for two days). He had an episode of focal seizure, four times with a mean duration of two minutes on the first and second postoperative days. Postoperative first day MRI confirmed the cerebellar hemorrhage (Figure 3).

A CT scan of the patient's head was obtained on postoperative day five and revealed a significant decrease in the cerebral and cerebellar edema, the previous surgery cavity reappeared and the cerebellar hematoma resembled the immediate postoperative brain CT. He required two antiepileptic combination treatments (Lamostrigine and phenytoin sodium). The patient had no seizure activity after starting the antiepileptic combinations. He had no significant neurological symptoms, and he was transferred to the ward on postoperative day five. He was discharged at day eight postoperatively, his postoperative follow-up history revealed that the patient had no seizure activity, and a neurologic examination was conducted on 12^th^ postoperative month.

## Discussion

Hemorrhage from the operative site is one of the most serious complications after intracranial surgery. But cerebellar hemorrhage after supratentorial intervention is a very infrequent complication. Remote cerebellar hematoma after supratentorial craniotomy is most common between the ages of 30 and 60 years [3]. This phenomenon is rare in children [5]. It was reported rare incidences ranging between 0.08 and 0.29% [6].We found only 6 cases of pediatric remote cerebellar hemorrhage complicating supratentorial craniotomy in literature (Table 1). With the difference of present case compared to the already published cases. The greater part of the previous cases are bilateral and asymmetrical. In contrast, the present case is unilateral. In addition 6 of 7 patients in the literature underwent for epilepsy surgery and lobectomy procedure was performed. Whereas presented case underwent a craniotomy to remove a moderate supratentorial dysembryoblastic neuroepithelial tumor. Therefore, primary disorders of present case and other cases are not same, but it obviously seems that a large cavity after removing of mass tumor resection or pronchymal tissue (lobectomy) may lead displacement of brain. However, in our case we observed remote cerebellar hemorrhage even there is no large cavity following tumor resection [6-11]. The most likely cause of cerebellar hemorrhage during supratentorial surgery is drainage of the cerebrospinal fluid (CSF) which results in the formation of a cerebellar “sag”. This mechanism causes transient occlusion of the superior bridging veins, which leads to hemorrhagic venous infarction [4,12]. This also plays a substantial role in the pathophysiological development of remote cerebellar hemorrhage.

The removal of a large, midline-localized supratentorial mass alone reduces intracranial pressure [4]. Excessive drainage of CSF causes intracranial hypotension, which may lead to a critical increase in the transluminal pressure of veins and the tearing of vessels [1,13,14].

The above factor does not necessarily explain the occurrence of this compliant after craniotomy to remove supratentorial mass in our case. As a matter, the incidence of this complication is too rare to be merely explained by the proposed factors, which are omnipresent in any neurosurgical case with CSF leak. If those were the basic etiology of this complication, the statistically expected incidence of this complication would be much higher. However, we believe that the above mechanisms explain the low possible physiopathologic mechanism of remote cerebellar hemorrhage. The mass was big and placed superficially in the left frontal lobe close to the midline. Therefore, during the operation, the basal cisterns, Sylvian fissure or ventricle were not opened and as a result, the CSF leakage was controlled. When cisterns are widely opened, this causes over drainage of CSF, resulting in decreased ICP [13].

We believe that a second factor was exaggerated dehydration. This was achieved by administration of an anti-edema agent as a diuretic (Forasemide) and 20% mannitol infusion. It is presumed that this aggressive intraoperative dehydration reduced the ICP, and consequently, sagging of the cerebellum might have reinforced the remote intra cerebral hematoma. Postoperative use of a subgaleal suction tube can lead to over drainage of the CSF, which may be playing a role in the displacement of the cerebellum, causing the stretching and tearing of the superior vermian veins and resulting in a remote cerebellar hematoma [6,13]. In the present case, we clearly performed hemostasis and we did not use a subgaleal drainage tube postoperatively.

Computed tomography is a superior diagnostic examination that is routinely used to determine acute intracranial occurrence [6].

The treatment principles can be divided into four categories;

1. The patient is conscious with moderate hemorrhage, no occlusive hydrocephalus, clinically is stable, and treatment strategy is conservative.
2. No brainstem compression, is occlusive hydrocephalus because of the fourth ventricle, ventricular drain is placed.
3. Brainstem is compressed, direct evacuation of hematoma by craniotomy.
4. Hemorrhagic involvement of both cerebellar hemispheres, the management is conservative [6].

In the present case, there was moderate hemorrhaging, but massive cerebellar and cerebral edema caused the patient to become somnolent. The patient was treated conservatively and the massive edema was resolved by the administration of an anti-edema agent.

In substance, we presumed over-dehydration during operation, and the removal of a large tumor in the midline were two factors that caused displacement of the cerebellum and intracranial hypotension. It is an understanding thing, CSF drainage and edema treatment are generally accepted principles of neuro-anesthesiology. It is widely applied every day in neurosurgery centers and again the incidence complication of remote cerebellar hemorrhage is extremely rare. Published reports in literature and presented case show that acute over leakage of CSF and exaggerated perioperative dehydration may predispose the patient to this complication.

Although dehydration and the displacement of the cerebellum are associated with this phenomenon after supratentorial surgery, the identification of the exact etiological factors remains elusive. It is advisable for case givers to be aware of the high potential risk of morbidity and mortality of this entity. Preoperative attention to prevent CSF overflow leakage and exaggerated dehydration of the patient may prevent remote cerebellar hemorrhages.

## List of abbreviations

CSF: Cerebrospinal fluid
CT: Computed Tomography
DNET: Dysembryoplastic Neuroepithelial Tumor
EEG: Electroencephalography
ICP: Intracranial pressure
MRI: Magnetic Resonance Imaging
